# Necrotizing Liver Granuloma/Abscess and Constrictive Aspergillosis Pericarditis with Central Nervous System Involvement: Different Remarkable Phenotypes in Different Chronic Granulomatous Disease Genotypes

**DOI:** 10.1155/2017/2676403

**Published:** 2017-01-10

**Authors:** Sanem Eren Akarcan, Neslihan Karaca, Guzide Aksu, Halil Bozkaya, Mehmet Fatih Ayik, Yasemin Ozdemir Sahan, Mehmet Arda Kilinc, Zafer Dokumcu, Cenk Eraslan, Emre Divarci, Hudaver Alper, Necil Kutukculer

**Affiliations:** ^1^Pediatric Immunology Department, Medical Faculty, Ege University, Izmir, Turkey; ^2^Interventional Radiology Department, Medical Faculty, Ege University, Izmir, Turkey; ^3^Cardiovascular Surgery Department, Medical Faculty, Ege University, Izmir, Turkey; ^4^Pediatric Cardiology Department, Medical Faculty, Ege University, Izmir, Turkey; ^5^Pediatric Intensive Care Unit, Medical Faculty, Ege University, Izmir, Turkey; ^6^Pediatric Surgery Department, Medical Faculty, Ege University, Izmir, Turkey; ^7^Radiology Department, Medical Faculty, Ege University, Izmir, Turkey; ^8^Pediatric Radiology Department, Medical Faculty, Ege University, Izmir, Turkey

## Abstract

Chronic granulomatous disease (CGD) is a primary immune deficiency causing predisposition to infections with specific microorganisms,* Aspergillus *species and* Staphylococcus aureus *being the most common ones. A 16-year-old boy with a mutation in CYBB gene coding gp91^phox^ protein (X-linked disease) developed a liver abscess due to* Staphylococcus aureus*. In addition to medical therapy, surgical treatment was necessary for the management of the disease. A 30-month-old girl with an autosomal recessive form of chronic granulomatous disease (CYBA gene mutation affecting p22^phox^ protein) had invasive aspergillosis causing pericarditis, pulmonary abscess, and central nervous system involvement. The devastating course of disease regardless of the mutation emphasizes the importance of early diagnosis and intervention of hematopoietic stem cell transplantation as soon as possible in children with CGD.

## 1. Introduction

Chronic granulomatous disease (CGD) is a primary immune deficiency in which NADPH oxidase function of phagocytes, responsible for respiratory burst and intracellular killing of microorganisms via reactive oxygen intermediates, is defective [1]. There is a predisposition to infections with certain microorganisms such as* Aspergillus, Staphylococcus aureus, Serratia marcescens, Burkholderia cepacia, Nocardia,* and* Salmonella *species [2–4].

Several mutations in genes encoding one of the five subunits of this enzyme complex were defined. A defect in gp91^phox^ component, encoded by CYBB gene, is the only X-linked (XL) form and the most common defect worldwide [2, 3, 5]. Defects in the other four components (p22^phox^, p47^phox^, p67^phox^, and p40^phox^) are due to autosomal recessive (AR) mutations (in CYBA, NCF1, NCF2, and NCF4 genes, resp.) [6]. Autosomal recessive forms are more frequently seen in countries where consanguineous marriages are prevalent [7, 8].

Age at diagnosis is usually earlier in XL form compared to AR forms giving clues about severity of illness [2, 3]. However, early and severe presentation may also be seen in AR patients [8]. Clinical outcome and manifestations can be quite different even in patients with similar mutations. Hereby, two cases with two different genetic defects and severe clinical presentations are presented to draw attention to hepatic, pericardial, and central nervous system involvements in CGD.

## 2. Case  1

A 16-year-old boy was referred to our hospital for symptoms of hepatosplenomegaly and pancytopenia with suspicion of metabolic diseases like Gaucher disease especially. Past medical history revealed cervical abscess in infantile period, an axillary abscess at the age of 10, and necrotizing pneumonia at the age of 14 years. He was the 6th child of nonconsanguineous parents with 10 live births. One brother deceased because of recurrent pulmonary infections.

Hepatosplenomegaly (spleen 10–12 cm, liver 8 cm below the costal margin), petechia on skin and mucous membranes, and decreased lung sounds were recorded on physical examination. Anemia, thrombocytopenia, neutropenia, hypergammaglobulinemia, and elevated ALP (alkaline phosphatase) (884 U/L) and GGT (gamma-glutamyl transferase) (356 U/L) levels were recorded as abnormal laboratory results. Chronic lung findings as bronchiectasis and atelectasis were observed in chest X-ray (CXR). Portal hypertension via portal Doppler US and biopsy proven perivenular fibrosis and congestion of liver were detected. Investigations revealed no significant results confirming any metabolic diseases including the early suspicion of Gaucher's disease. Pancytopenia was considered to be associated with splenomegaly related to portal hypertension and improved in follow-up. The quantitative oxidative burst activity (“Phagoburst” kit, Glycotope, Biotechnology) was abnormal (FMLP 0.94%, PMA 4.10%, and opsonized* E. Coli* 4.89%; normal values 1–10%, 98–100%, and 97–100%, resp.). He was diagnosed as CGD which was later confirmed with molecular analysis revealing a “hemizygous X-linked c.1609T>C mutation” in CYBB gene-gp91^phox^.

**Figure 1:**
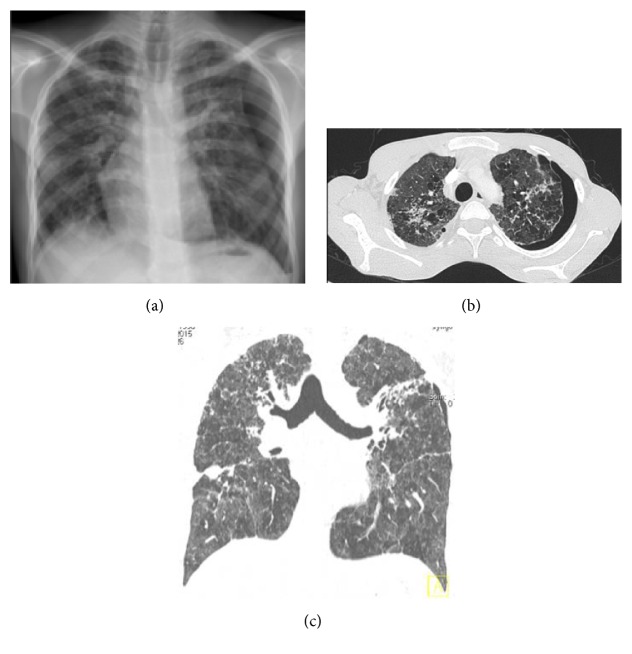
(a) Chest X-ray: Pneumothorax in left hemithorax, (b) and (c) thorax BT (sagittal and coronal sections): Bilateral chronic fibrotic changes with bronchiectasis and severe emphysema with air trapping in addition to left pneumothorax.

**Figure 2:**
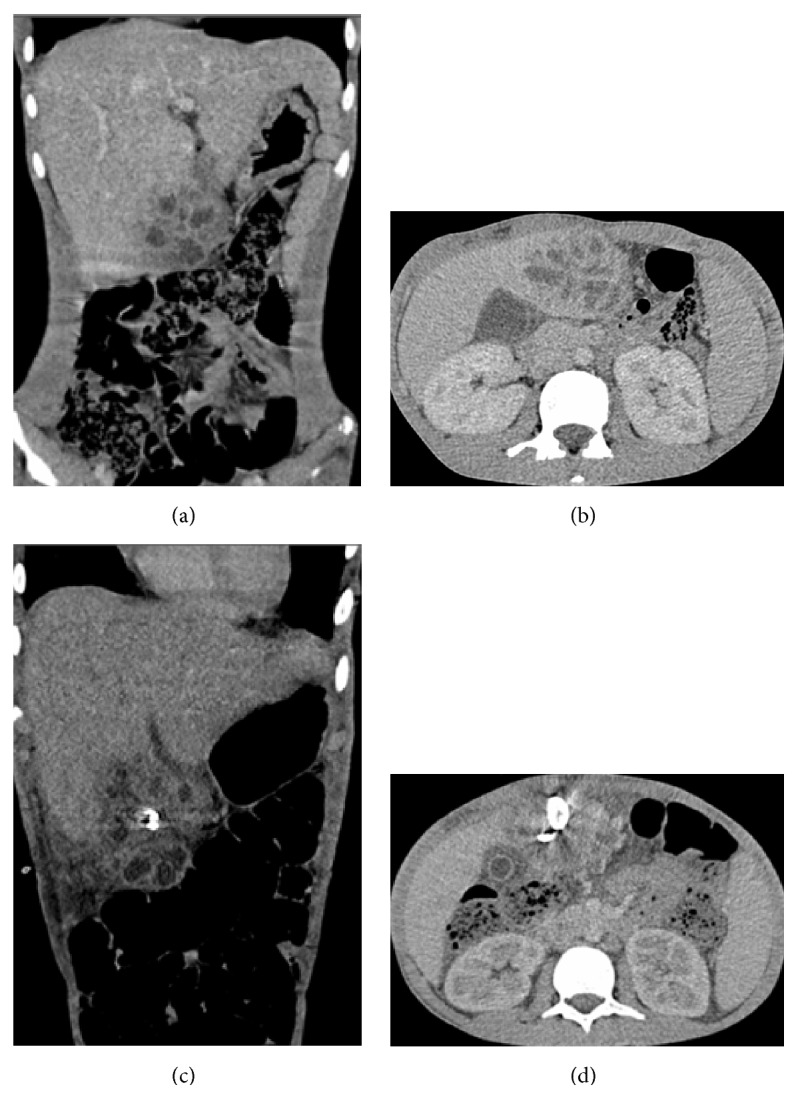
(a) and (b) Initial abdominal CT coronal and sagittal sections: A group of local collections in liver segment 4b consistent with abscesses in an area of 5 × 6.5 cm diameter; biggest one was 2.5 cm in diameter. Note that the lesion is extending outside the liver. (c) and (d) Control abdominal CT coronal and sagittal sections: After drainage catheter placement abscess did not drain due to excessive individual compartmentalization.

Prophylactic antifungal and antibacterial medications and interferon gamma-1b were started although patient adherence to the treatment and follow-up visits was very poor. He was admitted with the complaints of fever, cough, respiratory distress, and stomachache, two years after the diagnosis. He had bilateral fine crackles with decreased lung sounds in the left basal region, hepatosplenomegaly (spleen 2 cm, liver 3 cm palpable), and a swelling inferior to sternum with dimensions of 4 × 5 cm in abdomen. He had iron deficiency and chronic disease anemia, high acute-phase reactants (C-reactive protein (CRP) 11.2 mg/dL, erythrocyte sedimentation rate (ESR) 90 mm/hour), and minimally elevated ALP (133 U/L) and GGT (36 U/L) levels in laboratory evaluation. CXR revealed an expanded pneumothorax within left hemithorax. There were chronic lung findings relevant to recurrent infections in addition to pneumothorax in thorax CT (Figure 1). Ciprofloxacin, teicoplanin, and fluconazole were started empirically. There was a 7 × 4 cm heterogenous mass lesion in liver with cystic areas in center which was a multiloculated abscess in abdominal US that was subsequently confirmed with abdominal CT (Figure 2). There were multiple lymph nodes in periportal, peripancreatic, and para-aortic regions. Interventional radiology department inserted a drainage catheter to the abscess and a pigtail catheter to the left hemithorax.* Staphylococcus aureus *(methicillin sensitive) grew in the culture from abscess material. Antibiotics were replaced with cephazolin and rifampicin and a transient response was achieved. However, control CT 5 days after catheter placement showed no significant change in the dimensions of abscess (Figure 2). Surgical treatment was considered due to the multiloculated nature of the abscess and ongoing fever. Irregular abscess wall on anterior liver surface was dissected, and granulomatous changes in peripheral tissues were noted during the operation. Intraoperative US showed multiple small granulomas in liver. Fever did not recur followed by decreases in CRP to 1.4 mg/dL and in ESR to 22 mm/hr, after the operation. Pathologic findings were consistent with necrotizing granuloma and abscess in liver. He was discharged with supportive treatment and appropriate antibiotics two weeks after the operation. Hematopoietic stem cell transplantation (HSCT) was planned as the disease progressed despite the supportive measures. He had a full match donor but the family did not accept HSCT as a treatment option.

## 3. Case  2

A 3-month-old girl was referred to our hospital with a suspicion of Langerhans cell histiocytosis. She was hospitalized for pneumonia at the fifteenth day of birth followed by recurrent and treatment resistant cervical lymphadenopathies with high CRP levels. She was the first child of a consanguineous family. She had bilateral cervical lymphadenopathies and maculopapular skin eruptions over body and legs on admission. Laboratory investigations revealed anemia, leukocytosis, high CRP level (12 mg/dL), and hypergammaglobulinemia. Histological findings of the skin biopsy and lymph node excision were reported as granulomatous inflammation and necrotizing granulomatous lymphadenitis, respectively. Meanwhile, oxidative burst activity (“Phagoburst” kit, Glycotope, Biotechnology) was insufficient (FMLP 9%, PMA 9%, and opsonized* E. Coli* 4%; normal values 1–10%, 98–100%, and 97–100%, resp.) leading to a probable diagnosis of CGD later confirmed with mutation analysis disclosing a “homozygous autosomal recessive c.369 + 1G>A mutation” in CYBA gene-  p22^phox^.

**Figure 3:**
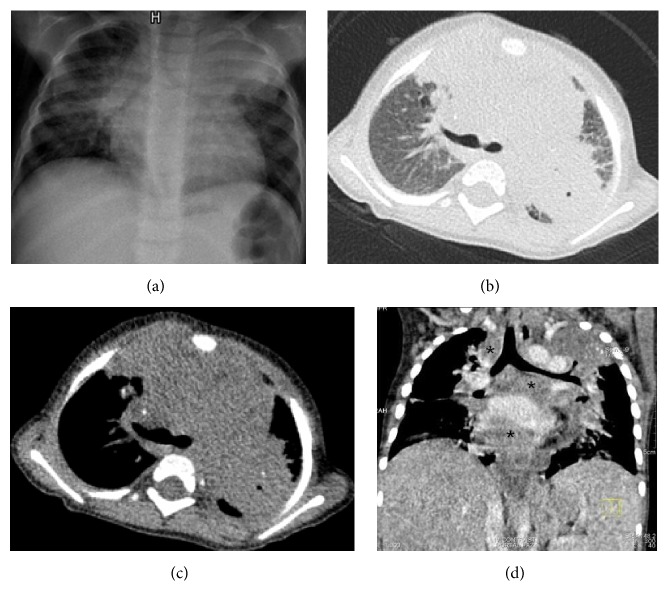
(a) Chest X-ray: Bilateral extensive infiltrations especially prominent in left upper lobe. (b) and (c) Thorax CT (parenchymal and mediastinal window, resp.): Right upper lobe anterior segment, left upper lobe nearly total consolidations. In particular the consolidation in left upper lobe and right paratracheal, hilar region includes calcifications. (d) Thorax CT (two months later, coronal section): Extensive visceral and parietal lympadenomegalies in mediastinum that reach 3 cm in diameter, some showing central necrosis especially the ones in pericardial neighborhood (*∗*).

She was admitted again with tachypnea with coarse lung sounds relevant to acute pneumonia at 30 months of age. She had anemia (Hb: 8.9 g/dL), leukocytosis (16800/mm^3^), and high CRP (3 mg/dL) and ESR (60 mm/hr) levels. CXR showed bilateral extensive infiltrations especially prominent in left upper lobe (Figure 3). Thorax CT revealed pneumonic consolidations with calcifications (Figure 3). These findings were interpreted in favor of tuberculosis (TB) although objective mycobacterial evidence was absent (PPD 9 mm, gastric fluid acid-fast bacilli and PCR negative). Three-drug combination therapy (isoniazid, rifampicin, and pyrazinamide) was initiated. Echocardiography showed pericardial thickening which was also evaluated as TB sequela.

**Figure 4:**
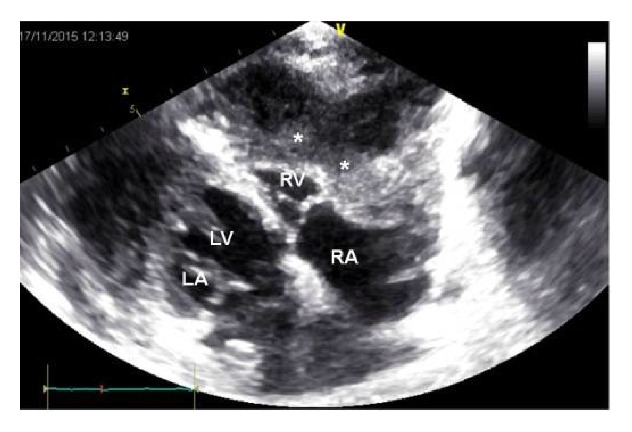
Echocardiography: Increased pericardial echogenity, pericardial thickening, localized liquid collections in apex adherent to pericardium (*∗*), and deviation of interventricular septum to left in inspiration (favoring pulmonary hypertension) and diastolic dysfunction due to constrictive pericarditis.

Despite treatment, her clinical condition worsened in a two-month period. Extensive lymph node enlargements in mediastinum and around pericardium reaching to 3 cm in diameter, some showing central necrosis, were recorded in the new thorax CT (Figure 3). During hospitalization, she developed right congestive heart failure signs, fine crackles in lungs, and increased oxygen need. Echocardiography showed findings compatible with pulmonary hypertension, diastolic dysfunction, and constrictive pericarditis (Figure 4). Emergency pericardiectomy was performed.* Aspergillus fumigatus *was cultured in pericardial fluid and blood* Aspergillus* antigen was positive for the first time. Parenteral caspofungin and voriconazole infusions were initiated in combination for antifungal therapy. Histological interpretation of the pericardial sample was reported as granulomatous pericarditis.

**Figure 5:**
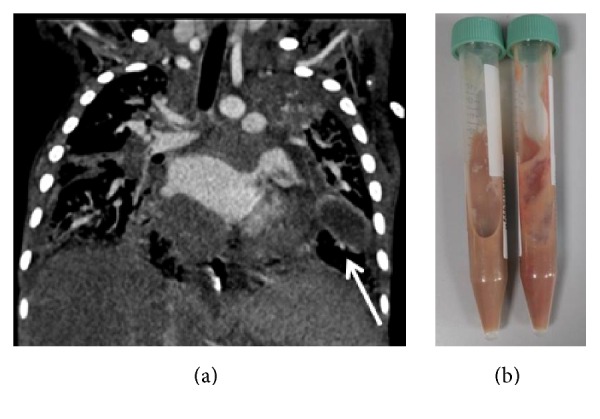
(a) Thorax CT coronal section: An abscess of 4.5 × 1.7 cm diameter in left inferior lobe in the neighborhood of cardiac apex (arrow). Parenchymal consolidations with punctate calcifications persevere. (b) Macroscopic appearance of the drainage material.

Fever and respiratory distress continued and thorax CT revealed an abscess of 4.5 × 1.7 cm diameter in left lung inferior lobe, two weeks after the pericardiectomy (Figure 5). The abscess was drained and drainage material culture was positive for* Aspergillus fumigatus *(Figure 5). Granulocyte transfusions were applied for the subsequent days and fever subsided gradually in a few days. She was discharged with oral voriconazole after her condition stabilized with parenteral antifungal therapy for one month. Blood* Aspergillus* antigen was still positive in low titers.

**Figure 6:**
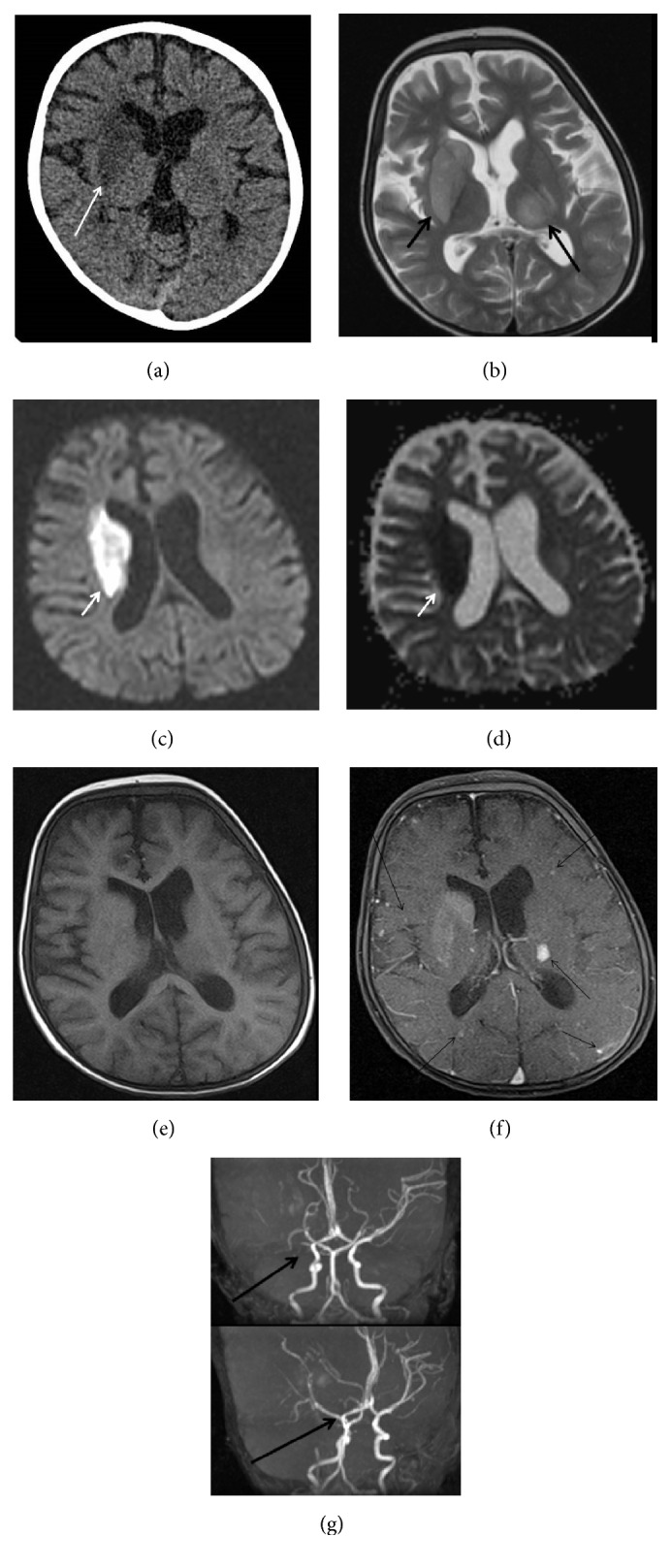
(a) Cranial CT: Hypodense area at the level of right basal ganglia compatible with acute ischemic infarction (arrow). (b) Cranial MR: Axial T2 image shows hyperintensity of right caudate nucleus, putamen, and globus pallidus. Contralateral thalamic hyperintensity is also seen (arrows). (c), (d) Diffusion restriction is seen at the level of right basal ganglia at diffusion b 1000 image and ADC map, respectively (arrow). (e), (f) Pre- and postcontrast T1 axial images show many enhancing intra-axial foci in both hemispheres after IV gadolinium administration (arrows). (g) Occlusion of right middle cerebral artery at the level of bifurcation on reconstructed MIP images of 3D TOF MR angiography.

Two weeks later, she was brought to emergency department with left hemiparesis, left central facial paralysis, and fever. Glasgow Coma Scale was 12, motor strength was 2/5 in left upper and lower extremities, and left plantar response was abnormal in neurological examination. A hypodense lesion at the level of right basal ganglia was interpreted as acute ischemic infarction in cranial CT (Figure 6). Diffusional restriction consistent with acute ischemia in right basal ganglia and cerebral, cerebellar millimetric enhancing foci consistent with aspergillosis were reported in cranial and diffusion magnetic resonance imaging (MRI) (Figure 6). In MR angiography, right middle cerebral artery was occluded from M2 segment to end which was also associated with* Aspergillus* infection (Figure 6). Voriconazole, amphotericin B, vancomycin, and meropenem were started. Intravenous immunoglobulin, 1 gr/kg/day, was given for two days. Fever was controlled and her general condition improved. Acetylsalicylic acid treatment was commenced to prevent further infarction.

**Figure 7:**
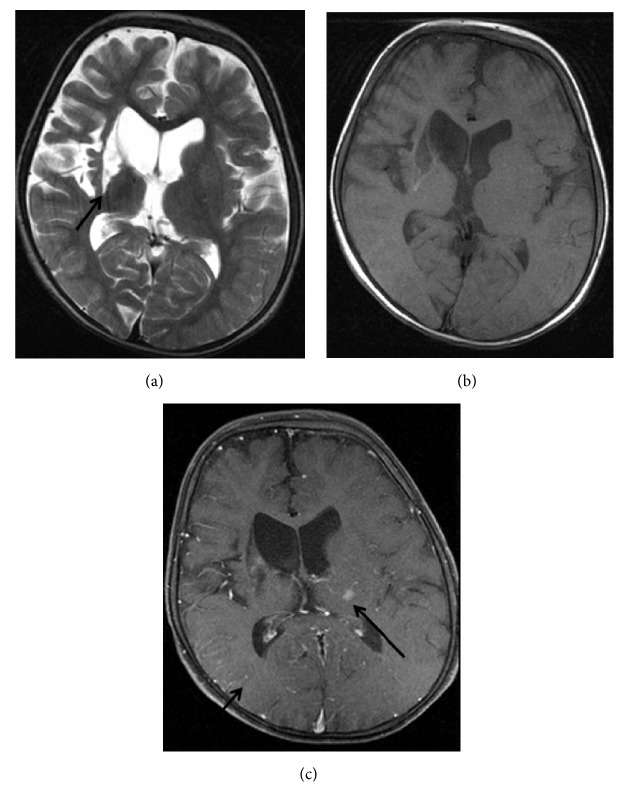
MR examination performed 5 months after the initial examination. (a) Axial T2 image shows the cystic encephalomalacia at the level of right basal ganglia (arrow). (b), (c) Partial resolution of enhancing foci is seen on pre- and postcontrast axial T1 images (arrows).

Neurological findings regressed with decreased facial asymmetry and increased motor strength in the follow-up. Parenteral antifungal treatment was continued for two months and she was discharged with oral voriconazole and negative blood* Aspergillus* antigen. The ischemic lesion in the right basal ganglia showed chronicity and appeared as cystic encephalomalacia; most of the cerebral/cerebellar foci were smaller than before and some disappeared in the control MRI five months after the initial evaluation (Figure 7). Due to the severe course of her disease, HSCT was planned and due to the lack of full matched relative, an unrelated donor search is initiated.

## 4. Discussion

Heterogeneous clinical presentations in CGD patients were attributed to the type of mutations in previous reports. XL-CGD patients had a smaller age at diagnosis compared to AR-CGD patients in two large cohorts from USA and Europe. Mean survival time and age at death were also higher in AR forms [2, 3]. These data were consistent with the general idea that AR-CGD had a milder clinical outcome. But in these cohorts, AR-CGD was rare (22–33%) compared to XL-CGD (67–70%) and p47^phox^ mutation was responsible in nearly half of AR-CGD patients [2, 3]. In an Iranian cohort, 87% of patients were AR-CGD (p47^phox^ mutation constituting 55%). The ages at onset of symptoms and diagnosis were lower in XL-CGD than AR-CGD. But when subgroups in AR-CGD were taken into account, patients with gp91^phox^ and p22^phox^ mutations were diagnosed significantly earlier than p47^phox^ mutations [7].

Recently, it was shown that the most important factor in severity of the disease and survival is the residual production of reactive oxygen intermediates (ROI) in neutrophils, not the mode of inheritance [1]. In a research from Turkey, AR-CGD was more common due to consanguinity and p22^phox^ mutation constituted 40% of them. Residual ROI production was present in all patients with p47^phox^ mutation and few patients with missense mutations in p22^phox^ and p67^phox^, but this was not the case for XL-CGD patients and remaining AR patients. Mean age at diagnosis of oxidase-residual patients was higher than that of oxidase-null patients [8].

Residual ROI production may be predicted by the specific mutation [1]. Our first case had a missense mutation in 537th amino acid of CYBB gene, defined as having normal gp91^phox^ protein expression (X91^+^) [5]. Missense mutations in amino acids 310 to 570 generally disrupt the enzymatic function without effecting protein expression. Many patients with gp91^phox^ missense mutations especially the ones effecting amino acids 1 to 309 had higher ROI production than nonsense, frameshift, splice, or deletion mutations [1]. Mutation in our patient may have caused a modest ROI production because he could survive to the age of 16 without diagnosis, prophylaxis, and good sanitary conditions although the symptoms started in the first year of life.

Second case had a splice site mutation in CYBA gene, causing absent p22^phox^ protein expression (A22°) [6]. ROI production in patients with p22^phox^ deficiency varies according to the mutation type, with missense mutations having the highest production [1]. Our patient had a very early presentation, persistent and frequent infections, and severe complications that were most probably related to very low or absent ROI production.

CGD causes characteristic recurrent infections and inflammatory complications in various organs, lung involvement reaching 80% of patients [2, 3, 9]. In a USA cohort,* Aspergillus *species were the most common isolated pathogens, in both pneumonia (41%) and lung abscesses (23%) [2]. Other less frequent infectious manifestations are pleural effusion, bronchiectasis, bronchitis, atelectasis, and mediastinal and hilar lymphadenopathy [10]. Chronic lung findings of Case  1 were related to recurrent and inadequately treated infections leading to his last admission with severe pneumothorax.

Inflammatory pulmonary manifestations such as granuloma formation, pneumonitis, and fibrosis may be seen in CGD patients [10]. Pulmonary granulomatous disease is diagnosed histologically with a negative culture and may be confused with mycobacterial infection [3]. Although not listed in the first five or six pathogens, mycobacterial infections due to* Mycobacterium tuberculosis* or BCG vaccination are not rare in CGD patients [11].

Case  2 had a previous lung biopsy performed for persistent lung findings and resulted as active chronic inflammation, micro abscess foci, inflammatory granulation, and fibrosis. She was given antituberculosis treatment for the recurring and persistent respiratory problems and calcifications on thorax CT but no response was achieved. All these findings were consistent with an early onset inflammatory lung involvement. The exact mechanism of abnormal inflammatory response and whether persistent or recurrent infections are triggering factors for inflammation in CGD are not clear. However, it is well known that fungi can cause tremendous inflammatory response in lungs [10]. Corticosteroids are recommended for inflammatory complications even in invasive infections [12]. We used low dose corticosteroid in Case  2 with limited improvement in respiratory symptoms.

Invasive fungal infection (IFI) is a major problem in nearly half of the CGD patients. Molds accounted for more than 60% of IFI with* Aspergillus *species especially* A. fumigatus* and A.* nidulans *causing 65% of mold infections [13, 14]. In 85% of cases, infection is limited to a single organ with the lung being the most common site (more than 95%) which is followed by bone and brain involvements each constituting only 10% of cases [14, 15].

*Aspergillus* pericarditis is not a frequent pathology and the firstly described cases were mostly immunocompromised patients with pulmonary aspergillosis diagnosed in autopsies [16]. In a review of 29 patients most having hematological malignancies with prolonged neutropenia, two CGD patients died although granulocyte transfusions in one and pericardiectomy in the other were performed in addition to antifungal therapy [17]. There is also a successfully treated case with long term use of voriconazole and caspofungin without surgery [18].

Case  2 had been receiving voriconazole already when symptoms of pericarditis emerged like most of the cases in the literature [17]. Her symptoms worsened under treatment followed by constrictive pericarditis. After surgery, caspofungin was added as second agent. A positive culture for* Aspergillus fumigatus* was obtained making this invasive fungal disease (IFD) episode a proven one [19]. Her respiratory distress and ongoing fever were most probably due to pulmonary aspergillosis causing dissemination to pericardium and leading to pericardial thickening.

Blood* Aspergillus* antigen is not a sensitive method to detect aspergillosis in CGD patients with a maximum sensitivity of 34% [13, 14]. But it may be useful in follow-up when found to be positive [20]. Blood* Aspergillus* antigen was found to be positive and remained positive for a long time in our patient after the identification of pericardial involvement. She also developed a lung abscess caused by* Aspergillus fumigatus* immediately after the pericardiectomy. The infection control was achieved by granulocyte transfusions besides abscess drainage and extensive antifungal therapy. Repeated granulocyte transfusions are increasingly used as a treatment choice when conventional therapies fail in life-threatening infections, especially with* Aspergillus* species [3, 14, 21–23].

Central nervous system (CNS) aspergillosis being an infrequent pathology is mostly seen in immune compromised patients. In more than 50% of cases, lungs or paranasal sinuses are primary foci of infection [24]. Hematogenous dissemination from lungs can cause meningitis, abscess formation, or vascular pathology in the form of thrombosis and infarction [25]. CNS aspergillosis constitutes 10% of IFD episodes in CGD patients, while it has a frequency of less than 5% in overall infections [14, 15]. Patients may present with seizures, headaches, fever, focal neurological signs per the pathology, and localization [14, 25]. Radiologically edema, hemorrhage, and ring enhancement are cardinal findings and nonspecific hyperintense foci are also defined [19, 24, 25].

Case  2 had a thrombus in right middle cerebral artery explaining her hemiparesis. She also had cerebral and cerebellar millimetric enhancing foci not causing significant symptoms. These lesions might be little foci not yet progressed to abscess formation. Amphotericin B and voriconazole both penetrating blood-brain-barrier were mostly used in combination with other antifungals in literature. Surgery is a treatment choice improving survival in patients with abscesses but was not necessary in our case [15, 24–26]. After two months of parenteral antifungal treatment, oral voriconazole was planned to be continued for at least 12–18 months for this patient.

Liver involvement in CGD is a frequent finding. Transient or persistent liver enzyme abnormalities, liver abscesses, hepatosplenomegaly, portal hypertension, granuloma, and hepatitis in biopsy specimens are among the most common findings [27]. In a recent cohort, 32% developed liver abscess and in 47% of them, abscesses recurred. The most common isolated pathogen was* S. aureus* (86%) [4]. Liver abscesses are dense and generally have multiple loculations complicating percutaneous drainage and mostly require surgery [28, 29]. In Case  1, hepatomegaly was a consistent finding for a long period. He had also significant ALP and GGT elevations, portal hypertension, and hypersplenism findings at the time of diagnosis. The presence of multiple granulomas reported in the abscess drainage confirmed with biopsy was an expected finding in CGD [27]. Hepatomegaly without liver enzyme anomalies was also a finding in Case  2 even before constrictive pericarditis. Therefore, a patient with hepatic enlargement and relevant history should be suspected for CGD and be examined for other symptoms of the disease.

In conclusion, CGD patients may have very severe and life-threatening infections and complications such as hepatic, pericardial, and central nervous system involvement. The management and treatment of these patients are too difficult, risky, and expensive. Therefore, early diagnosis and consequent hematopoietic stem cell transplantation in early childhood period before serious organ damage takes place are of utmost importance for CGD cases, both AR and XL forms, as in severe combined immune deficiency patients.
